# THE HEALTH OF DIVERSE CALIFORNIANS WITH NEEDS FOR LONG-TERM SERVICES AND SUPPORTS

**DOI:** 10.1093/geroni/igae098.3173

**Published:** 2024-12-31

**Authors:** Kathryn Kietzman, Lei Chen, Preeti Juturu

**Affiliations:** University of California Los Angeles, Los Angeles, California, United States; University of California San Francisco, San Francisco, California, United States; University of California Los Angeles, Los Angeles, California, United States

## Abstract

This study used data from the 2019-2020 California Long-Term Services and Supports (CA-LTSS) survey to identify differences in health outcomes within a population of older adults and adults with disabilities. Our study sample included 2030 adults who lived in home and community settings in California, and experienced at least one type of LTSS difficulty, including cognitive impairment and difficulties performing activities of daily living and/or instrumental activities of daily living. We conducted chi-square tests using weighted representative California population-level data. Among people with LTSS difficulties, we found significant racial/ethnic differences in health insurance coverage, usual source of care, and psychological distress. For example, a larger proportion of Asian adults with LTSS difficulties reported having no health insurance (9.0%), having no usual source of care (21.2%), and experiencing serious psychological distress (37.3%) compared to other racial/ethnic groups. We also found significant differences by employment status. Specifically, adults with LTSS difficulties who were in the labor market were more likely to experience serious psychological distress (28.8%), have no health insurance (11.3%), experience delays in receiving care or prescriptions (26.1%), have unmet needs (41.1%), and report financial worries (68.7%), compared to adults with LTSS difficulties who were not in the labor market. Income level was also significantly associated with health and well-being, health insurance coverage, usual source of care, and unmet needs. These results can be used to inform the development of programs and policies for diverse people with LTSS needs, and further suggest the need for innovative strategies to advance health equity.

